# Pangenome analysis reveals genetic isolation in *Campylobacter hyointestinalis* subspecies adapted to different mammalian hosts

**DOI:** 10.1038/s41598-021-82993-9

**Published:** 2021-02-09

**Authors:** Daniela Costa, Simon Lévesque, Nitin Kumar, Pablo Fresia, Ignacio Ferrés, Trevor D. Lawley, Gregorio Iraola

**Affiliations:** 1 Microbial Genomics Laboratory, Institut Pasteur Montevideo, 11400 Montevideo, Uruguay; 2 Sección Genética Evolutiva, Facultad de Ciencias, Universidad de la República, Montevideo, Uruguay; 3 Laboratoire de Santé Publique du Québec, Quebec City, Canada; 4 Wellcome Sanger Institute, Hinxton, Cambridgeshire UK; 5 Unidad Mixta UMPI, Institut Pasteur de Montevideo + Instituto Nacional de Investigación Agropecuaria INIA, Montevideo, Uruguay; 6 Center for Integrative Biology, Universidad Mayor, Santiago de Chile, Chile

**Keywords:** Computational biology and bioinformatics, Evolution, Microbiology

## Abstract

*Campylobacter hyointestinalis* is an emerging pathogen currently divided in two subspecies: *C. hyointestinalis* subsp. *lawsonii* which is predominantly recovered from pigs, and *C. hyointestinalis* subsp. *hyointestinalis* which can be found in a much wider range of mammalian hosts. Despite *C. hyointestinalis* being reported as an emerging pathogen, its evolutionary and host-associated diversification patterns are still vastly unexplored. For this reason, we generated whole-genome sequences of 13 *C. hyointestinalis* subsp. *hyointestinalis* strains and performed a comprehensive comparative analysis including publicly available *C. hyointestinalis* subsp. *hyointestinalis* and *C. hyointestinalis* subsp. *lawsonii* genomes, to gain insight into the genomic variation of these differentially-adapted subspecies. Both subspecies are distinct phylogenetic lineages which present an apparent barrier to homologous recombination, suggesting genetic isolation. This is further supported by accessory gene patterns that recapitulate the core genome phylogeny. Additionally, *C. hyointestinalis* subsp. *hyointestinalis* presents a bigger and more diverse accessory genome, which probably reflects its capacity to colonize different mammalian hosts unlike *C. hyointestinalis* subsp. *lawsonii* that is presumably host-restricted. This greater plasticity in the accessory genome of *C. hyointestinalis* subsp. *hyointestinalis* correlates to a higher incidence of genome-wide recombination events, that may be the underlying mechanism driving its diversification. Concordantly, both subspecies present distinct patterns of gene families involved in genome plasticity and DNA repair like CRISPR-associated proteins and restriction-modification systems. Together, our results provide an overview of the genetic mechanisms shaping the genomes of *C. hyointestinalis* subspecies, contributing to understand the biology of *Campylobacter* species that are increasingly recognized as emerging pathogens.

## Introduction

The genus *Campylobacter* consists in a diverse group of bacteria currently classified into 32 species and 13 subspecies. Among them, *C. jejuni* and *C. coli* have drawn most of the attention because they are leading causes of human gastroenteritis worldwide^1^. However, the recent application of whole-genome sequencing to study bacterial populations has increased the clinical awareness of campylobacteriosis and highlighted the importance of other neglected *Campylobacter* species, like *C. fetus*^2–5^, as causative agents of human and animal infections. Among them, *C. hyointestinalis* is an emerging pathogen that was first isolated from swine with proliferative enteritis^6^ and has since been sporadically recovered from human infections, but also found as a commensal in a wide variety of wild, farm and domestic mammals (including cattle, pigs, dogs, hamsters, deer and sheep)^7^.

*C. hyointestinalis* is currently divided in two subspecies based on genetic and phenotypic traits^8,9^. While *C. hyointestinalis* subsp. *hyointestinalis* has a broad host range, *C. hyointestinalis* subsp. *lawsonii* has been predominantly recovered from pigs. Some pioneering studies at both genetic and protein levels have suggested that *C. hyointestinalis* harbors even further intraspecific diversity^10–12^ which could facilitate its adaptation to diverse hosts and environments. However, these observations remain to be assessed at higher resolution due to the lack of available genomic data for both subspecies. So, the evolutionary forces driving their genetic and ecological distinctions have not been explored at the whole-genome level.

Here, we performed whole-genome sequencing of 13 *C. hyointestinalis* subsp. *hyointestinalis* strains isolated from healthy cattle and from a natural watercourse, that were collected from farms located around Sherbrooke, Québec, Canada. By incorporating this information to the available genomes of both subspecies, we performed a pangenome analysis to elucidate the main sources of molecular diversity in both subspecies and the probable genetic mechanisms and functional characteristics that distinguish the presumably host-restricted *C. hyointestinalis* subsp. *lawsonii* from the generalist *C. hyointestinalis* subsp. *hyointestinalis*. Our work provides the first comparative analysis of both *C. hyointestinalis* subspecies at the pangenome level and will guide future efforts to understand the patterns of host-associated evolution in emerging *Campylobacter* pathogens.

## Results

By whole-genome sequencing 13 *C. hyointestinalis* subsp. *hyointestinalis* strains, we enlarged by 45% the current collection of genomes available for *C. hyointestinalis*. Then, by recovering 29 additional genomes of *C. hyointestinalis* subsp. *hyointestinalis* (n = 19) and *C. hyointestinalis* subsp. *lawsonii* (n = 10) from public databases, we built a genomic dataset consisting of 42 genomes (Table 1). These genomes represent strains isolated between 1985 and 2016 from five different hosts in six different countries. This dataset was used to apply comparative pangenomic, phylogenetic and ecological approaches to uncover the main sources of genetic variability between *C. hyointestinalis* subspecies.

**Table 1:** Information of *C. hyointestinalis* genomes analyzed in this work.

Strain	Subspecies	Country	Date	Host	Material	Reference
006A-0059	Chh	Canada	2006	Cow	Feces	This study
006A-0063	Chh	Canada	2006	Cow	Feces	
006A-0073	Chh	Canada	2006	Cow	Feces	
006A-0091	Chh	Canada	2007	Cow	Feces	
006A-0113	Chh	Canada	2007	Cow	Feces	
006A-0161	Chh	Canada	2007	Cow	Feces	
006A-0170	Chh	Canada	2007	Cow	Feces	
006A-0178	Chh	Canada	2007	Cow	Feces	
006A-0180	Chh	Canada	2007	Cow	Feces	
006A-0191	Chh	Canada	2007	Cow	Feces	
006A-0193	Chh	Canada	2007	Cow	Feces	
006A-0196	Chh	Canada	2007	Cow	Feces	
007A-0283	Chh	Canada	2005	-	Freshwater	
S1563d	Chh	New Zealand	2016	Cow	Feces	Wilkinson et al. (2018)^15^
S1564d	Chh	New Zealand	2016	Cow	Feces	
S1509d	Chh	New Zealand	2016	Cow	Feces	
S1501d	Chh	New Zealand	2016	Cow	Feces	
S1597b	Chh	New Zealand	2016	Sheep	Feces	
S1603d	Chh	New Zealand	2016	Cow	Feces	
S1559c	Chh	New Zealand	2016	Cow	Feces	
S1599c	Chh	New Zealand	2016	Cow	Feces	
VP28b	Chh	New Zealand	2010	Deer	Feces	
VP26b	Chh	New Zealand	2008	Deer	Feces	
VP28	Chh	New Zealand	2009	Deer	Feces	
VP30b	Chh	New Zealand	2011	Deer	Feces	
S1499c	Chh	New Zealand	2016	Cow	Feces	
S1614a	Chh	New Zealand	2016	Cow	Feces	
S1592a	Chh	New Zealand	2016	Cow	Feces	
S1547c	Chh	New Zealand	2016	Sheep	Feces	
DSM 19053	Chh	United States	1985	Pig	Intestine	NCBI
ATCC 35217	Chh	United States	1985	Pig	Intestine	JGI
LMG-9260	Chh	Belgium	1986	Human	Feces	Miller et al. (2016)^43^
CCUG-27631	Chl	Sweden	1990	Pig	Stomach	
RM10074	Chl	United States	2009	Pig	NA	Bian et al. (2018)^44^
RM9767	Chl	United States	2009	Pig	NA	
RM9004	Chl	United States	2009	Pig	NA	
RM10071	Chl	United States	2009	Pig	NA	
RM9752	Chl	United States	2009	Pig	NA	
RM9426	Chl	United States	2009	Pig	NA	
RM10075	Chl	United States	2009	Pig	NA	
RM14416	Chl	NA	1988	Cow	Feces	
CHY5	Chl	United Kingdom	NA	Pig	Stomach	

### Genetic diversity of *C. hyointestinalis *subsp.* hyointestinalis* strains sequenced in this study

To determine the degree of genetic variability among the new *C. hyointestinalis* subsp. *hyointestinalis* genomes generated from strains isolated in Canada, we used the currently available multilocus sequence typing (MLST) scheme for *C. hyointestinalis*. This analysis revealed that 7 out of 13 (54%) genomes presented new sequence types (STs). Out of them, three new STs (strains 006A-0063, 006A-0178 and 006A-0196) were product of new combinations of previously described alleles. The remaining novel STs were product of previously unknown alleles for genes *tkt*, *aspA*, *glnA* and *pgm*. Remarkably, not a single *C. hyointestinalis* subsp. *hyointestinalis* genome sequenced in this study harbored the same MLST genotype (Table S1).

### *C. hyointestinalis* subspecies are genetically isolated lineages

**Figure 1:**
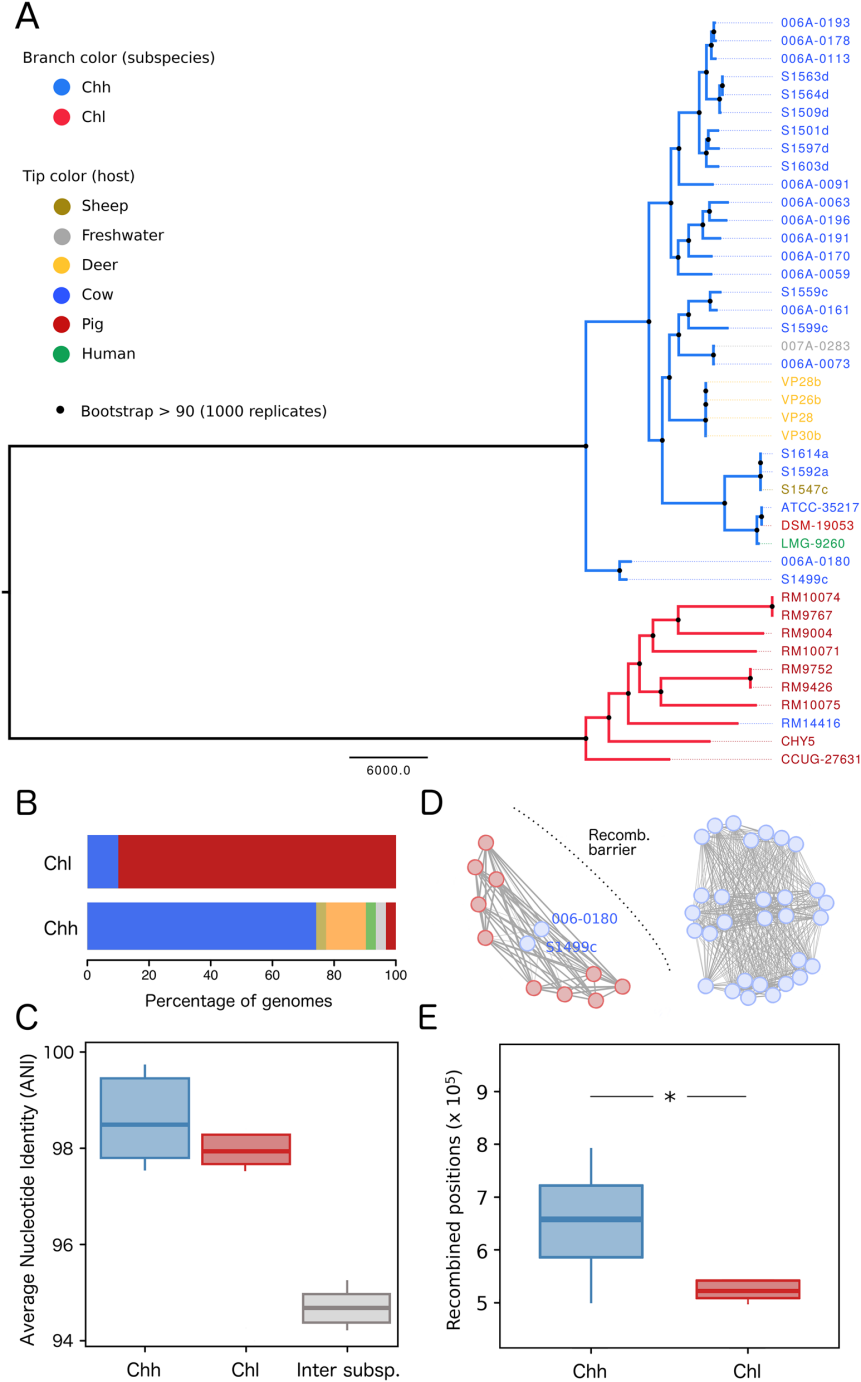
Phylogeny and recombination of ecologically distinct *C. hyointestinalis* subspecies. **(A)** Core genome phylogeny of species *C. hyointestinalis*. Branch color highlights the *C. hyointestinalis* subsp. *lawsonii* lineage in red and *C. hyointestinalis* subsp. *hyointestinalis* in blue. Tip labels indicate strain name and are colored according to isolation source. Branch lengths expressed in number of substitutions is showed in the bottom of the tree. **(B)** Barplot showing the distribution of hosts in both *C. hyointestinalis* subspecies. **(C)** Boxplots showing ANI values calculated within and between genomes belonging to each subspecies. Inter-subspecies ANI is around 95%, suggesting both subspecies are close to the standard boundary for species definition. **(D)** Network analysis of shared recombinant blocks (edges) between *C. hyointestinalis* genomes (vertexes). Any pair of genomes is connected with an edge if they share any recombinant block. Edge width is proportional to the number of recombinant blocks shared by genome pairs. A recombination barrier is evidenced between *C. hyointestinalis* subsp. *hyointestinalis* and *C. hyointestinalis* subsp. *lawsonii.* **(E)** Boxplots showing the number of recombined positions in the genomes of both subspecies. A statistically significant differences is observed in favor of *C. hyointestinalis* subsp. *hyointestinalis* (p = 0.0035, Mann–Whitney U test).

To gain insight into the population structure of *C. hyointestinalis* we reconstructed the species clonal phylogeny starting from a core genome alignment that consisted in 1,320,272 positions (representing 66% of the longest genome). After removing recombinations only 81,000 positions (representing 6% of the original core genome alignment) remained in the clonal frame. The resulting clonal phylogeny showed a highly structured topology with both subspecies completely separated in two distinct lineages with clear differences in host distribution (Fig. 1A,B). This was in line with a mean Average Nucleotide Identity (ANI)^13^ of ~ 95% separating *C. hyointestinalis* subsp. *hyointestinalis* from *C. hyointestinalis* subsp. *lawsonii* (Fig. 1C). Evidence supporting the genetic isolation of both subspecies also came from exploring genome-wide recombination patterns, which revealed a barrier to homologous recombination between *C. hyointestinalis* subsp. *hyointestinalis* from *C. hyointestinalis* subsp. *lawsonii* (with the exception of *C. hyointestinalis* subsp. *hyointestinalis* strains S1499c and 006A-0180 that have recombined with *C. hyointestinalis* subsp. *lawsonii* strains) (Fig. 1D). Furthermore, *C. hyointestinalis* subsp. *hyointestinalis* seems to be much more recombinogenic than *C. hyointestinalis* subsp. *lawsonii,* as evidenced by a significantly higher proportion of their genomes contained within recombinant regions (Fig. 1E).

### Accessory genes discriminate both *C. hyointestinalis* subspecies

**Figure 2:**
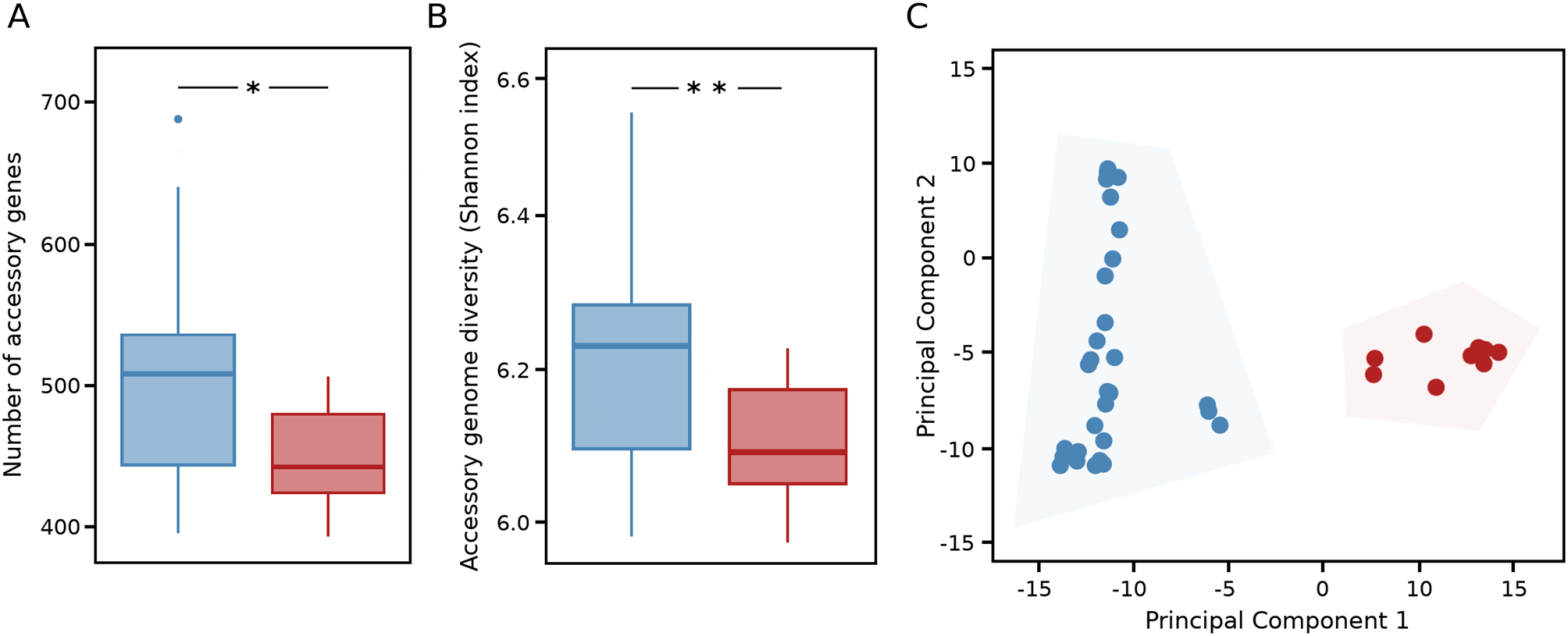
Distinct accessory genomes in *C. hyointestinalis* subspecies. **(A)** Boxplots showing the number of accessory genes (accessory genome size) in both subspecies. *C. hyointestinalis* subsp. *hyointestinalis* possesses a slightly significantly bigger accessory genome than *C. hyointestinalis* subsp. *lawsonii* (p = 0.023, Mann–Whitney U test). **(B)** Boxplots showing the diversity of accessory genes (as measured by the inverted Simpson index) in both subspecies. *C. hyointestinalis* subsp. *hyointestinalis* has a significantly more diverse accessory genome than *C. hyointestinalis* subsp. *lawsonii* (p = 0.00021, Mann–Whitney U test). **(C)** Principal component analysis using accessory gene patterns showing that both subspecies represent two completely distinct clusters.

To gain further insight into the genomic evolution of *C. hyointestinalis* subspecies we reconstructed its pangenome. A total of 4317 gene clusters were identified out of which 3040 (70%) were accessory genes (Table S2). The accessory genome median size was 580 (IQR = 174) and 538 (IQR = 74) for *C. hyointestinalis* subsp. *hyointestinalis* and *C. hyointestinalis* subsp. *lawsonii*, respectively. Figure 2A shows a slightly significant difference in the accessory genome size in favor of *C. hyointestinalis* subsp. *hyointestinalis* (p = 0.023, Mann–Whitney U test). To discard possible confounding effects due to the unbalanced number of genomes available for each subspecies, we repeated this analysis by sub-sampling *C. hyointestinalis* subsp. *hyointestinalis* genomes to the number of available *C. hyointestinalis* subsp. *lawsonii* genomes. This analysis revealed a still observable difference in the accessory genome size in favor of *C. hyointestinalis* subsp. *hyointestinalis* (Fig. S1). This tendency was also observable when calculating the diversity of accessory genes using the inverted Simpson’s index for both subspecies (p = 0.00021, Mann–Whitney U test) (Fig. 2B). Accessory gene presence/absence patterns also allowed to completely discriminate between *C. hyointestinalis* subsp. *hyointestinalis* and *C. hyointestinalis* subsp. *lawsonii* using a Principal Components Analysis (PCA), indicating that they have subspecies-specific accessory gene repertories (Fig. 2C). Indeed, 1562 accessory gene clusters were exclusively found in *C. hyointestinalis* subsp. *hyointestinalis* genomes while only 618 were specific to *C. hyointestinalis* subsp. *lawsonii* genomes.

### Functional distinctions in the accessory genome of *C. hyointestinalis* subspecies

**Figure 3:**
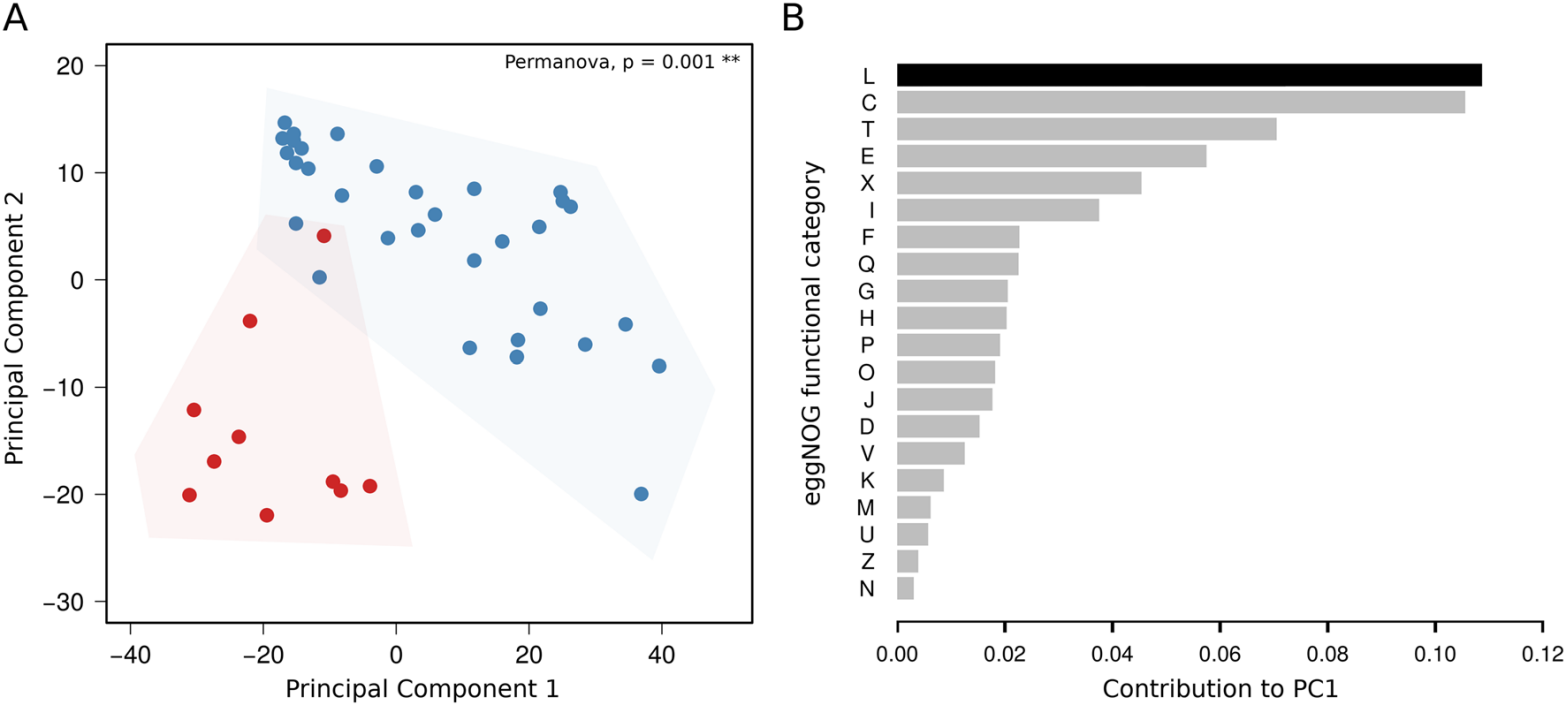
Functionally distinct accessory genomes in *C. hyointestinalis* subspecies. **(A)** Principal component analysis showing that *C. hyointestinalis* subspecies form two different clusters (p = 0.001, Permanova test) based on the functional analysis of their accessory genes. **(B)** Boxplot showing the contribution of each functional category to the variance explained by the first principal component (PC1). Functional category codes resemble those used by the eggNOG database. The top-ranking category (L: recombination and DNA repair) is highlighted in black.

**Figure 4:**
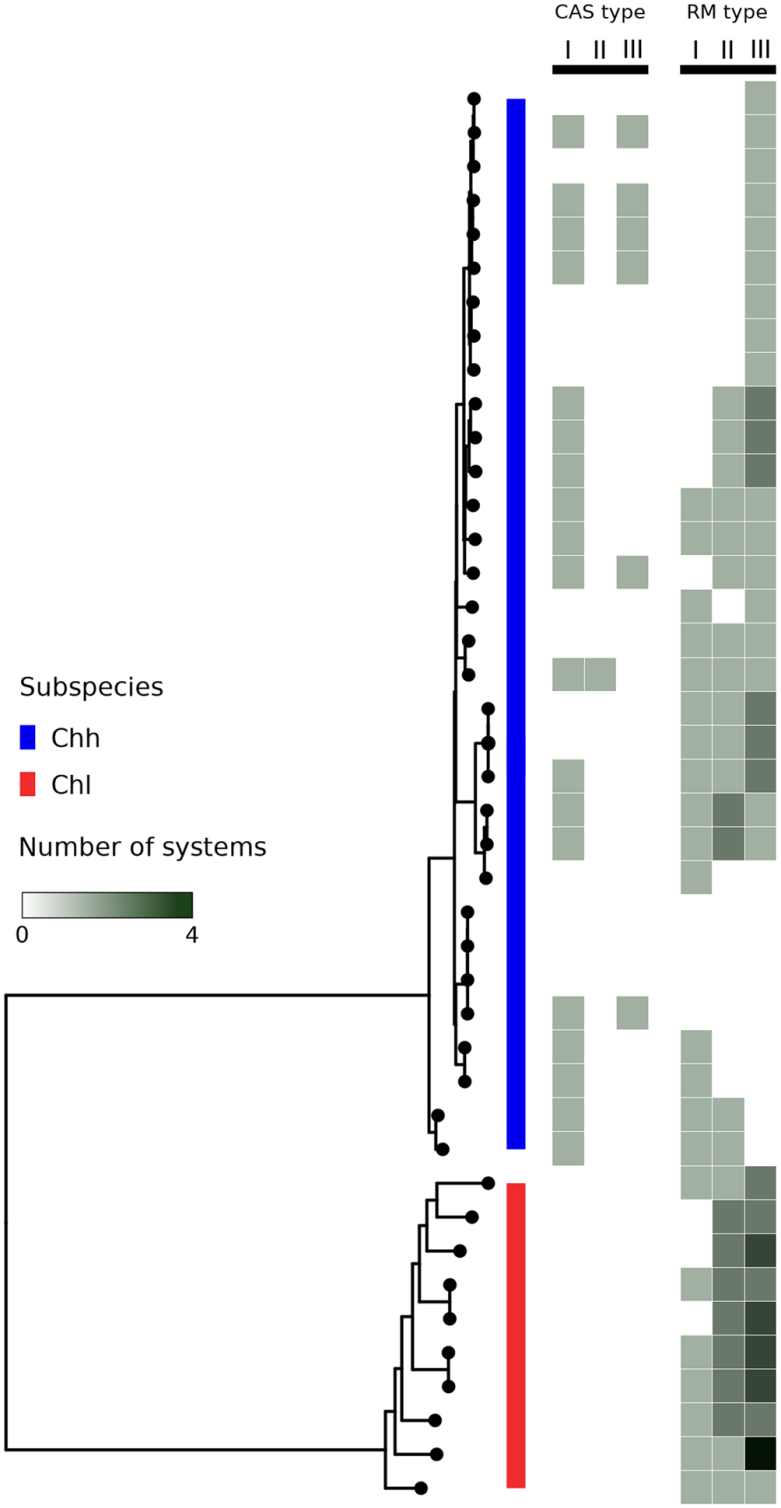
Different repertories of CRISPR/Cas proteins and R-M systems between subspecies. Phylogenetic tree of *C. hyointestinalis* subspecies annotated with information CRISPR/Cas systems and R-M systems. Vertical strips in the right side are colored according to copy number of complete CRISPR/Cas and R-M systems classified by type. Detailed information about copy number is provided in Table S4.

To evaluate possible functional aspects associated to different accessory gene patterns distinguishing *C. hyointestinalis* subspecies, we performed a functional classification based on the eggNOG database^14^. First, we found a complete separation of subspecies when using functional annotations to perform a PCA (p = 0.001, Permanova test), supporting that accessory genomes are functionally different between them (Fig. 3A). Then, we looked for functional categories that could discriminate between subspecies and we found that genes belonging to the functional category referred as “DNA replication, recombination and repair” (L) presented the most informative discriminatory patterns (Fig. 3B). Given this evidence, we studied CRISPR-associated proteins (Cas) and Restriction-Modification (R-M) systems, which are known to be involved in DNA recombination and repair. Figure 4 shows that Cas systems are more diverse and widespread in *C*. *hyointestinalis* subsp. *hyointestinalis*. Importantly, our analysis did not find any complete Cas system in C. *hyointestinalis* subsp. *lawsonii* genomes. In particular, CAS system type I was the most prevalent in *C. hyointestinalis* subsp. *hyointestinalis* genomes (59%) A higher number of complete R-M systems were found in *C. hyointestinalis* subsp. *lawsonii* (mean = 5), than in *C. hyointestinalis* subsp. *hyointestinalis* (mean = 2). In particular, type II and type III R-M systems had > 2 copies in 90% of *C. hyointestinalis* subsp. *lawsonii* genomes and only in 25% of *C. hyointestinalis* subsp. *hyointestinalis* genomes.

## Discussion

Recently, the first analysis of multiple *C. hyointestinalis* strains confirmed the previously observed highly diverse nature of this bacterial species at the whole-genome level^15^ by comparing strains recovered from ruminant livestock in New Zealand. Despite these strains being collected in geographically close locations, they showed all different and novel MLST genotypes. Similarly, our collection of isolates from Canada also presented different MLST genotypes (most of which were also novel), suggesting that patterns of high genomic variability in *C. hyointestinalis* are a distinctive feature of this species in geographically distant populations.

Hitherto, main patterns of variation in *C. hyointestinalis* have been identified by studying *C. hyointestinalis* subsp. *hyointestinalis* genomes. This limitation prevented to compare if the observed trends were conserved between both subspecies or if evolutionary forces are differentially impacting their genomes. Accordingly, not only our work increased the availability of *C. hyointestinalis* subsp. *hyointestinalis* genomes from a previously unsampled geographic region, but also took advantage of the recent release of novel *C. hyointestinalis* subsp. *lawsonii* genomes to perform a comparative pangenome analysis that revealed the main forces underpinning the genomic diversity now considering both subspecies.

Despite sampling bias may exist, the available data indicate that *C. hyointestinalis* subspecies are ecologically distinct, given that *C. hyointestinalis* subsp. *lawsonii* has been mainly recovered from pigs while *C. hyointestinalis* subsp. *hyointestinalis* is a generalist that colonizes several mammalian species. Host specialization has been observed in other *Campylobacter* species, such as in *C. fetus* lineages that preferably infect cows, humans or reptiles^3,16^, in phylogenetically distinct *C. coli* isolates from diseased humans or riparian environments^17^, and in global clonal complexes of *C. jejuni* with differential host preferences^18^. In most of these cases, strong lineage-specific recombination and accessory gene gain/loss patterns have been identified, concordantly to what is expected for bacterial lineages that undergo ecological isolation. For example, a barrier to homologous recombination like that observed between *C. hyointestinalis* subspecies has been also detected between mammal- and reptile-associated *C. fetus* subspecies^16^, and lineage-specific recombination patterns have been found in the *C. jejuni* clonal complex ST-403 that is unable to colonize chicken^19^. Interestingly, this is correlated with the presence of lineage-specific repertories of R-M systems, as well as we observed for type II and type III R-M systems in *C. hyointestinalis* subspecies. Moreover, other molecular mechanisms involved in genome plasticity like CRISPR/Cas systems are unevenly distributed in agricultural or non-agricultural *C. jejuni*/*coli* genomes^20^, indicating that these systems are differentially present in ecologically distinct niches resembling again the patterns we observed for type I Cas systems in *C. hyointestinalis* subspecies. However, the presence of two *C. hyointestinalis* subsp. *hyointestinalis* genomes (006A-0180 and S1499c) of cattle origin recombining with *C. hyointestinalis* subsp. *lawsonii* genomes of porcine origin, suggests that both subspecies have occupied the same niche at some point either in cattle or pigs. This had been previously observed for strain S1499c by Wilkinson et al. (2018)^15^, which is defined by the authors as an atypical isolate with a particularly highly diverse genome. Interestingly, strains 006A-0180 and S1499c form a divergent branch within the *C. hyointestinalis* subsp. *hyointestinalis* clade indicating they are genetically distinct. This suggests the probable existence of yet unsampled intermediate lineages between genetically isolated *C. hyointestinalis* subsp. *hyointestinalis* and *C. hyointestinalis subsp. lawsonii* that have kept the capacity of exchanging genetic material. Also, the available genomic dataset for *C. hyointestinalis* subsp. *lawsonii* is mostly composed by strains isolated in the same geographic region (California, United States). This may be a confounding factor given that conclusions about the genetic isolation of this subspecies could change if a more diverse dataset is included. Additionally, a more diverse set of *C. hyointestinalis* subsp. *hyointestinalis* genomes are needed to confirm this observation, in particular isolated for strains isolated from pigs. This would allow to investigate genetic relatedness between both subspecies coexisting in the same host. Alternatively, the observed recombination barrier could not be entirely related to genomic differences preventing horizontal gene transfer and recombination between subspecies, and might be explained by the physical isolation of both subspecies in different hosts.

The maintenance of lineage-specific repertories of molecular machineries that modulate genome plasticity is probably an extended mechanism in *Campylobacter*, considering that recombination is an important evolutionary force for the adaptation and acquisition of a host signature in well-known *Campylobacter* pathogens^21^. In general, adaptation occurs in favor of gradual host specialization, but generalism is also widely observed in nature, for example in extremely successful *C*. *jejuni* lineages that can be found in high prevalence from both agricultural sources or human infections^22^. A generalist phenotype can be thought as an advantage for bacteria that colonize farm animals, since it allows the subsistence in multiple mammalian species that thieve in close proximity. However, this also represents an increased risk for zoonotic transmission since these animals are usually in contact with humans. Indeed, this scenario is reflected in *C. hyointestinalis* subspecies, given that the generalist *C. hyointestinalis* subsp. *hyointestinalis* has been frequently isolated from human infections in contrast to the lack of reported cases of human infections with *C. hyointestinalis* subsp. *lawsonii.*

Despite our analysis uncovered the main forces shaping the intra-specific diversity of *C. hyointestinalis* and our results support the observed epidemiological pattern in both subspecies, the availability of comprehensive genomic datasets for most campylobacters is quite restricted^23^, so the integration of strain collections from different hosts, geographic regions and clinical conditions is necessary to deepening our understanding of the genomic evolution in this emerging pathogen and other neglected *Campylobacter* species.

## Methods

### Sampling and bacterial isolation

Samples were collected as described previously^24^. Briefly, cattle feces samples were transported in Enteric Plus medium (Meridian Bioscience Inc, Ohio, USA) and processed on the same day. About 1–2 g of each fecal sample were transferred to 25 ml of Preston selective enrichment broth (Oxoid, Nepean, Ontario, Canada) and incubated 3–4 h at 37 ºC and then transferred to 42 ºC and incubated for 48 h. After incubation, 20 μl were streaked on a Karmali plate (Oxoid) and incubated at 42 ºC for 48 h. For environmental water, 3000 ml of water were collected and transported on ice to the laboratory, held at 4 ºC and tested within 24 h. Water was filtered through a 0.45 μm pore-size membrane filter and Preston broth and Karmali plate were used as above to isolate *Campylobacter*.

### Whole genome sequencing, available data and taxonogenomic analyses

Cells were pelleted from culture plates and phosphate-buffered saline (PBS). Genomic DNA preparation was performed using a BioRobot M48 (Qiagen). DNA was prepared and sequenced using the Illumina Hi-Seq platform with library fragment sizes of 200–300 bp. and a read length of 100 bp at the Wellcome Sanger Institute. Each sequenced genome was de novo assembled with Velvet^25^, SSPACE v2.0^26^ and GapFiller v1.1^27^ with default parameters. Resulting contigs were annotated using Prokka^28^. Species membership was checked by calculating the Average Nucleotide Identity (ANI) index as previously described^29^. Multilocus sequence typing (MLST) was performed from genomic assemblies with the available *C. hyointestinalis* scheme at PubMLST (https://www.pubmlst.org) using MLSTar^30^. Available genomic data at the time of designing this work consisted in 19 *C. hyointestinalis* subsp. *hyointestinalis* strains and 10 *C. hyointestinalis* subsp. *lawsonii* strains, that were added to the 13 *C. hyointestinalis* subsp. *hyointestinalis* sequenced in this work (Table S3) to build a final dataset of 42 genomes (Table 1).

### Pangenome and recombination analyses

A multiple genome alignment was performed with the progressiveMauve algorithm^31^ and the final core genome alignment was defined by concatenating locally collinear blocks (LCBs) longer than 500 bp present in at least 41 out of 42 genomes (~ 98%). Recombinant regions were identified running Gubbins^32^ with default parameters. The pan-genome was reconstructed using Pewit (https://github.com/iferres/pewit)^33^. Briefly, for every genome, each annotated gene is scanned against the Pfam database^34^ using HMMER3 v3.1b2 hmmsearch^35^ and its domain architecture is recorded (presence and order). A primary set of orthologous clusters is generated by grouping genes sharing exactly the same domain architecture. Then, remaining genes without hits against the Pfam database are compared to each other at protein level using HMMER3 v3.1b2 phmmer and clustered using the MCL algorithm^36^. These coarse clusters are then splitted using a tree-pruning algorithm which allows to discriminate between orthologous and paralogous genes. This algorithm automatically aligns each gene cluster and builds a Neighbor-Joining tree and iteratively refines coarse clusters and detects paralogous using each gene-tree to i) detect nodes whose descendants all belong to the same genome and ii) split the tree in many subtrees as necessary to achieve the minimum set of subtrees with just one tip per genome. Finally, singletons (genes occurring in a single genome) generated in the previous steps are refined by trying to reallocate them to previously generated clusters by comparing each singleton against cluster-specific HMMs using HMMER3 v3.1b2 hmmsearch^35^. Accessory genes were defined as those gene clusters occurring in less than 98% of the genomes (41/42). Ecological distances over accessory gene patterns like Jaccard index (to measure diversity between strains) or Shannon index (to measure intra-genomic diversity) and Principal Component Analysis (PCA) were calculated with the base or vegan^37^ packages in R v3.6.0.

### Analysis of specific gene families and functional categories

Several specific gene families of interest were recovered and analyzed form *C. hyointestinalis* genomes. CRISPR-associated protein (CAS) gene clusters were identified and classified using CRISPRCasFinder^38^. To recover R-M systems we followed the methodology described in Oliveira et al. (2016)^39^. This approach identifies systems by searching genes encoding MTase and REase components from the REBASE database^40^ using Blast + blastp^41^ with an identity > 80% and query coverage > 80% as inclusion thresholds. Complete R-M systems of each type were considered if MTase and REase components were less than four genes apart each other. Functional categories were assigned to annotated genes using the egg NOG database^14^ and the eggNOG-mapper tool^42^.

## Supplementary Information

Supplementary Figure S1.

Supplementary Table S1.

Supplementary Table S2.

Supplementary Table S3.

Supplementary Table S4.
