# Supplementary figures and images for: Pangenome analysis reveals genetic isolation in Campylobacter hyointestinalis subspecies adapted to different mammalian hosts
Source: Sci Rep. 2021 Feb 9;11:3431. doi: 10.1038/s41598-021-82993-9 (PMC7873201; doi:10.1038/s41598-021-82993-9)

Number of accessory genes

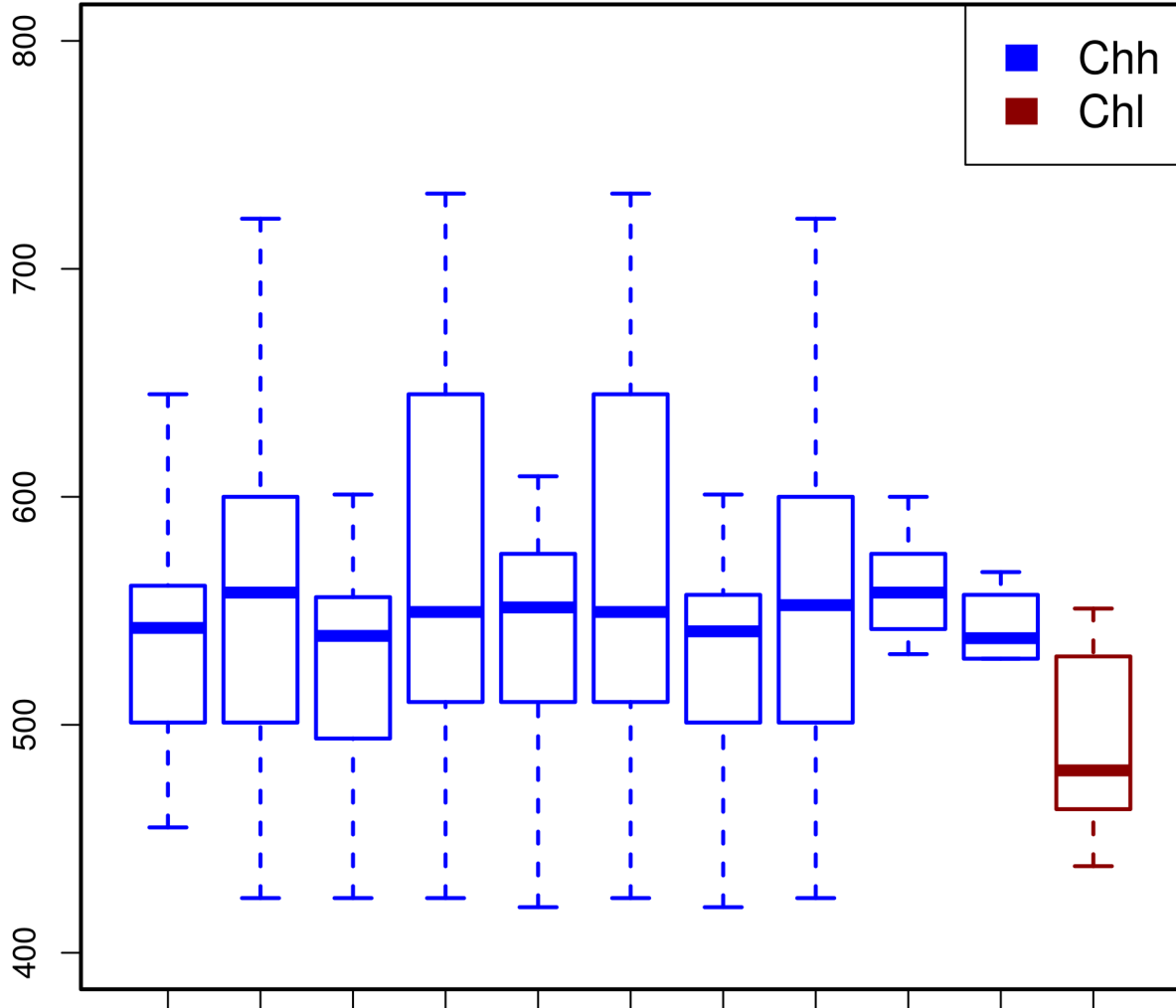

Supplement: Supplementary file 1 — Supplementary Figure S1. [file 41598_2021_82993_MOESM1_ESM.pdf]
